# Efficient strategies for leave-one-out cross validation for genomic best linear unbiased prediction

**DOI:** 10.1186/s40104-017-0164-6

**Published:** 2017-05-02

**Authors:** Hao Cheng, Dorian J. Garrick, Rohan L. Fernando

**Affiliations:** 1 Department of Animal Science, Iowa State University, Ames, 50011 Iowa USA; 2 Department of Statistics, Iowa State University, Ames, 50011 Iowa USA; 3 Institute of Veterinary, Animal & Biomedical Science, Massey University, Palmerston North, New Zealand

**Keywords:** Leave-one-out cross validation, GBLUP

## Abstract

**Background:**

A random multiple-regression model that simultaneously fit all allele substitution effects for additive markers or haplotypes as uncorrelated random effects was proposed for Best Linear Unbiased Prediction, using whole-genome data. Leave-one-out cross validation can be used to quantify the predictive ability of a statistical model.

**Methods:**

Naive application of Leave-one-out cross validation is computationally intensive because the training and validation analyses need to be repeated n times, once for each observation. Efficient Leave-one-out cross validation strategies are presented here, requiring little more effort than a single analysis.

**Results:**

Efficient Leave-one-out cross validation strategies is 786 times faster than the naive application for a simulated dataset with 1,000 observations and 10,000 markers and 99 times faster with 1,000 observations and 100 markers. These efficiencies relative to the naive approach using the same model will increase with increases in the number of observations.

**Conclusions:**

Efficient Leave-one-out cross validation strategies are presented here, requiring little more effort than a single analysis.

## Background

A random multiple-regression model that simultaneously fit all allele substitution effects for additive markers or haplotypes as uncorrelated random effects was proposed for Best Linear Unbiased Prediction (BLUP) [1], using whole-genome data. Breeding values are defined as the sum of the effects of all the markers or haplotypes, and their estimates are widely used for prediction of the merit of selection candidates. Estimates of marker or haplotype effects are used to predict breeding values of individuals that were not present in a previous analysis commonly referred to as training. An alternative earlier published approach to use marker or haplotype information fits breeding values as random effects based on covariances defined by a “genomic relationship matrix” computed from genotypes [2]. These two models have been shown to be equivalent in terms of predicting breeding values [3, 4] and we refer to them here as marker effect models (MEM) or breeding value models (BVM), the latter often known as Genomic Best Linear Unbiased Prediction (GBLUP).

Cross validation is often used to quantify the predictive ability of a statistical model. In k-fold cross validation, the whole dataset is partitioned into k parts with k analyses, where one part is omitted for training with validation on the omitted part. Leave-one-out cross validation (LOOCV) is a special case of k-fold cross validation with *k*=*n*, the number of observations. When the dataset is small, leave-one-out cross validation is appealing as the size of the training set is maximized. However, naive application of LOOCV is computationally intensive, requiring *n* analyses.

We show below how LOOCV can be performed using either the MEM or BVM with little more effort than is required for a single analysis with *n* observations.

## Methods

Use of the MEM is more efficient when the number *n* of individuals is larger than the number *p* of markers, because for this model the mixed model equations are of order *p* plus the number of other effects. When *n*<*p*, estimated breeding values can be obtained more efficiently by solving the mixed model equations for the BVM of order *n* plus the number of other effects. We deal with the special case where the only other effect is a general mean and phenotypes have been pre-corrected for other nuisance variables. Efficient strategies for LOOCV using this special case for MEM when *n*≥*p* and BVM when *p*≥*n* are shown below.

### Marker effect models

The MEM for GBLUP can be written as

1 $$ \boldsymbol{y}=\mathbf{1}\mu+\boldsymbol{X}\boldsymbol{\beta}+\boldsymbol{e},  $$

where ***y***, a *n*×1 vector for phenotypes, has been pre-corrected for all fixed effects other than *μ*, the overall mean, ***X*** is the *n*×*p* matrix of marker covariates, $\mathcal {\boldsymbol {\beta }}$ is a *p*×1 random vector of the allele substitution effects and ***e*** is a *n*×1 random vector of residuals. Often it is assumed that marker effects are identically and independently distributed (iid) random variables with null means and variances $\sigma _{\beta }^{2}$. Thus, under the usual assumption that the residuals are iid with null means and variances $\sigma _{e}^{2}$, *E*(**y**)=**1** *μ*. When MEM is used, LOOCV can be performed by using a well-known strategy used in least-squares regression to compute the predicted residual sum of square (PRESS) [5] statistic.

#### LOOCV strategy for MEM

BLUP of $\mathcal {\boldsymbol {\beta }}^{*}=\left [ \begin {array}{c} \mathbf {\mu }\\ \boldsymbol {\beta } \end {array}\right ]$ can be obtained by solving the mixed model equations

2 $$ \left(\boldsymbol{X}^{*{\prime}}\boldsymbol{X}^{*}+\boldsymbol{D}\lambda\right)\hat{\boldsymbol{\beta}^{*}}=\boldsymbol{X}^{*{\prime}}y,  $$

where ***X*** ^∗^= [ **1** ***X***], ${\hat {\boldsymbol {\beta }^{*}}}=\left [ \begin {array}{c} \hat {\mathbf {\mu }}\\ \hat {\mathbf {\boldsymbol {\beta }}} \end {array}\right ]$, ***D*** is a diagonal matrix whose elements are 0 followed by a *p* vector of 1s and $\lambda =\frac {\sigma _{e}^{2}}{\sigma _{\beta }^{2}}$.

Now, BLUP for ${\boldsymbol {\beta }_{-j}^{*}}$, where observation *j* is left out, can be obtained as

3 $$ {\hat{\boldsymbol{\beta}}^{*}_{-j}}=\left(\boldsymbol{X}^{*{\prime}}_{-j}\boldsymbol{X}^{*}_{-j}+\boldsymbol{D}\lambda\right)^{-1} \boldsymbol{X}^{{*{\prime}}}{~}_{-j}\boldsymbol{y}_{-j},  $$

where $\boldsymbol {X}_{-j}^{*}$ is ***X*** ^∗^ with the *j*th row removed and ***y***_−*j*_ is ***y*** with the *j*th element removed.

Suppose $\boldsymbol {x}^{*{\prime }}_{j}$ is the *j*th row of ***X*** ^∗^, then from the matrix inverse lemma [4],

4 $$ {\begin{aligned} {}\left(\boldsymbol{X}^{*{\prime}}_{-j}\boldsymbol{X}^{*}_{-j}+\boldsymbol{D}\lambda\right)^{-1} & =\left(\boldsymbol{X}^{*{\prime}}\boldsymbol{X}^{*}+\boldsymbol{D}\lambda-\boldsymbol{x}^{*}_{j}\boldsymbol{x}^{*{\prime}}_{j}\right)^{-1}\\ & =\left(\boldsymbol{X}^{*{\prime}}\boldsymbol{X}^{*}+\boldsymbol{D}\lambda\right)^{-1}\\ &\quad-\frac{\left(\boldsymbol{X}^{*{\prime}}\boldsymbol{X}^{*} +\boldsymbol{D}\lambda\right)^{-1}\!\boldsymbol{x}^{*}_{j}\boldsymbol{x}^{*{\prime}}_{j}\left(\boldsymbol{X}^{*{\prime}}\boldsymbol{X}^{*} \,+\,\boldsymbol{D}\lambda\right)^{-1}}{1-H_{jj}}, \end{aligned}}  $$

where the quadratic $H_{jj}=\boldsymbol {x}^{*}_{j'}\left (\boldsymbol {X}^{*{\prime }}\boldsymbol {X}^{*}+\boldsymbol {D}\lambda \right)^{-1}\boldsymbol {x}^{*}_{j}$ is the *j*th diagonal element of ***H***=***X*** ^∗^(***X*** ^∗′^ ***X*** ^∗^+***D*** *λ*)^−1^ ***X*** ^∗′^.

Using (3) in (4), the prediction residual for the *j*th observation can be written as

5 $$\begin{array}{*{20}l} {}\hat{e_{j}} & =y_{j}-\boldsymbol{x}^{*{\prime}}_{j}\hat{\boldsymbol{\beta}^{*}}_{-j} \\ & =y_{j}-\boldsymbol{x}^{*{\prime}}_{j}\left[{\vphantom{\frac{1}{2}}}\left(\boldsymbol{X}^{*{\prime}}\boldsymbol{X}^{*}+\boldsymbol{D}\lambda\right)^{-1}\right. \\ &\quad\left.-\frac{\left(\boldsymbol{X}^{*{\prime}}\boldsymbol{X}^{*}\,+\,\boldsymbol{D}\lambda\right)^{-1}\!\boldsymbol{x}^{*}_{j}\boldsymbol{x}^{*{\prime}}_{j} \left(\boldsymbol{X}^{*{\prime}}\boldsymbol{X}^{*}\,+\,\boldsymbol{D}\lambda\right)^{-1}}{1-H_{jj}}\right]\!\boldsymbol{X}^{*{\prime}}_{-j}\boldsymbol{y}_{-j} \\ & =\frac{\left(1-\boldsymbol{H}_{jj}\right)y_{j}-\boldsymbol{x}^{*{\prime}}_{j}\left(\boldsymbol{X}^{*{\prime}}\boldsymbol{X}^{*} +\boldsymbol{D}\lambda\right)^{-1}\boldsymbol{X}^{*}_{-j'}\boldsymbol{y}_{-j}}{1-\boldsymbol{H}_{jj}} \end{array} $$

6 $$\begin{array}{*{20}l} & =\frac{y_{j}-\boldsymbol{x}^{*{\prime}}_{j}\hat{\boldsymbol{\beta}^{*}}}{1-\boldsymbol{H}_{jj}}. \end{array} $$

These prediction errors can be squared and accumulated over *n* realizations to compute PRESS defined as $\sum _{j=1}^{n}\hat {e_{j}}^{2}$. The accuracy of genomic prediction is often quantified as the correlation between the predicted and observed values of *y*_*j*_, and that correlation can be estimated from the values of $\hat {y}_{j}$, which can be computed efficiently as $\hat {y}_{j}=y_{j}-\hat {e}_{j}$, using the observed values of *y*_*j*_. When a specific group of individuals is of interest, prediction accuracies and PRESS can also be calculate using $\hat {e_{j}}$ for individuals in that group.

### Breeding value models

When *n*<*p*, the genomic prediction of the breeding value $\mathbf {x}_{j}'\hat {\mathbf {\boldsymbol {\beta }}}$ can be obtained more efficiently by solving the mixed model equations for the BVM:

7 $$\begin{array}{*{20}l} \boldsymbol{y} & =\mathbf{1}\mu+\mathbf{Z}\mathbf{u}+\boldsymbol{e}, \end{array} $$

where **u**=**X** **β**, ${\text {var}\left (\mathbf {u}\right)=\mathbf {XX'}\sigma _{\beta }^{2}}$, **Z** is the identity matrix of order *n* and other variables are as in the MEM. Further, in both models E(**y**)=**1** *μ*, and $\text {var}(\mathbf {y})=\mathbf {X}\mathbf {X}'\sigma _{\beta }^{2}+\mathbf {I}\sigma _{e}^{2}$. These two models are said to be equivalent [6], and linear functions predicted from one model are identical to corresponding predictions from the other model. Two efficient strategies for LOOCV using the BVM are shown below.

#### LOOCV strategy I for BVM

The mixed model equations for this model are:

8 $$ \left(\boldsymbol{Z}^{*{\prime}}\boldsymbol{Z}^{*}+\boldsymbol{G}\lambda\right)\hat{\boldsymbol{u}^{*}}=\boldsymbol{Z}^{*{\prime}}y,  $$

where $\boldsymbol {Z}^{*}=[\!\mathbf {1}~~ \boldsymbol {Z}],\boldsymbol {\hat {u}}^{*}=\left [ \begin {array}{c} \hat {\mathbf {\mu }}\\ \hat {\mathbf {\boldsymbol {u}}} \end {array}\right ], \mathbf {G}=\left [ \begin {array}{cc} 0 & \mathbf {0^{'}}\\ \mathbf {0} & \left (\boldsymbol {XX'}\right)^{-1} \end {array}\right ]$. Due to the relative order of the coefficient matrices for the MEM and the BVM, when *n*<*p*, $\boldsymbol {x}^{*{\prime }}_{j}\hat {\boldsymbol {\beta }^{*}}$ is more efficiently obtained as $\hat {u^{*}}_{j}$. Similarly, $\text {var}(\boldsymbol {x}^{*{\prime }}_{j}\boldsymbol {\beta }^{*}-\boldsymbol {x}^{*{\prime }}_{j}\hat {\boldsymbol {\beta }^{*}})=\mathbf {x^{*{\prime }}}_{j} \left (\mathbf {X}^{*{\prime }}\mathbf {X}^{*}+\mathbf {D}\lambda \right)^{-1}\mathbf {x}^{*}_{j}\sigma _{e}^{2}$ can be obtained more efficiently as $\text {var}(u_{j}^{*}-\hat {u^{*}}_{j})=\mathbf {z^{*}}_{j'}\left (\mathbf {Z}^{*{\prime }}\mathbf {Z^{*}}+\mathbf {G}\lambda \right)^{-1} \mathbf {z^{*}}_{j}\sigma _{e}^{2}$. Using these two equalities, the formula for $\hat {e}_{j}$ becomes:

9 $$\begin{array}{*{20}l} \hat{e_{j}} & =y_{j}-\boldsymbol{x}^{*{\prime}}_{j}\hat{\mathbf{\boldsymbol{\beta}^{*}}}_{-j} \\ & =\frac{y_{j}-\boldsymbol{x}^{*{\prime}}_{j}\hat{\mathbf{\boldsymbol{\beta}^{*}}}}{1-H_{jj}} \\ & =\frac{y_{j}-\boldsymbol{x}^{*{\prime}}_{j}\hat{\mathbf{\boldsymbol{\beta}^{*}}}}{1-\boldsymbol{x}^{*{\prime}}_{j}\left(\mathbf{X}^{*{\prime}} \mathbf{X}^{*}+\mathbf{D}\lambda\right)^{-1}\mathbf{x}^{*}_{j}} \\ & =\frac{y_{j}-\mathbf{z}^{*{\prime}}_{j}\hat{u^{*}}}{1-\mathbf{z}^{*{\prime}}_{j}\left(\mathbf{Z}^{*{\prime}}\mathbf{Z}^{*}+\mathbf{G}\lambda\right)^{-1} \mathbf{z}^{*}_{j}} \\ & =\frac{y_{j}-\mathbf{z}^{*{\prime}}_{j}\hat{u}^{*}}{1-\boldsymbol{C}_{jj}}, \end{array} $$

where the quadratic $\mathbf {z}^{*{\prime }}_{j}\left (\mathbf {Z}^{*{\prime }}\mathbf {Z}^{*}+\mathbf {G}\lambda \right)^{-1}\mathbf {z}^{*}_{j}$ is the *j*th diagonal element of ***C***=***Z*** ^∗^(***Z*** ^∗′^ ***Z*** ^∗^+***G*** *λ*)^−1^ ***Z*** ^∗′^.

#### LOOCV strategy II for BVM

Another efficient strategy for BVM is shown here. First we consider the situation where ***y*** has been pre-corrected for *μ* in addition to nuisance effects so that E(**y**)=**0** and we define $\mathbf {var\left (\boldsymbol {y}\right)=X}\mathbf {X}'\sigma _{\beta }^{2}+\mathbf {I}\sigma _{e}^{2}=\boldsymbol {V}$. Now matrix ***Q*** is constructed by augmenting the covariance matrix of ***y*** with one leading row and column as

$$\mathbf{Q}=\left[ \begin{array}{cc} \mathbf{y'y} & \mathbf{y^{{\prime}}}\\ \mathbf{y} & \mathbf{V} \end{array}\right]. $$

To obtain the prediction error for observation *j*, the second row and column of **Q** are permuted with row and column *j*+1. In this manner **Q** has its rows and columns symmetrically permuted as **P** *j*′**Q** **P**_*j*_ ***=W***, where the permutation matrix **P**_*j*_ is obtained by permuting the second row of the *n* order identity matrix with row *j*+1. So the permuted matrix is:

$$\mathbf{W}=\left[\begin{array}{ccc} \mathbf{y'y} & y_{j} & \mathbf{y}_{-j}^{{\prime}}\\ y_{j} & V_{jj} & \mathbf{V}_{j,-j}\\ \mathbf{y}_{-j} & \mathbf{V}_{-j,j} & \mathbf{V}_{-j,-j} \end{array}\right]=\left[ \begin{array}{cc} \mathbf{\mathbf{A}} & \mathbf{\mathbf{B}}\\ \mathbf{\mathbf{B^{'}}} & \mathbf{\mathbf{C}} \end{array}\right] $$

where we will define the leading 2×2 matrix as $\mathbf {A}=\left [ \begin {array}{cc}\mathbf {y'y} & y_{j}\\ y_{j} & V_{jj} \end {array}\right ]$, and the other partitions as $\mathbf {B}=\left [ \begin {array}{c}\mathbf {y}_{-j}^{{\prime }}\\ \mathbf {V}_{j,-j} \end {array}\right ]$, and **C**=**V**_−*j*,−*j*_, where −*j* denotes that the *j*th element, row or column has been removed. Defining *W* ^11^ as the top left or leading 2×2 sub-matrix in *W* ^−1^ corresponding to the position of **A** in **W**, and using partitioned inverse-matrix identities [7], the inverse of *W* ^11^ can be written as,

10 $$\begin{array}{*{20}l} {}\left(\mathbf{W^{11}}\right)^{-1} & =\mathbf{A}-\mathbf{B}\mathbf{C^{-1}}\mathbf{B^{{\prime}}} \\ & =\left[ \begin{array}{cc} \boldsymbol{y}'\boldsymbol{y} & y_{j}\\ y_{j} & V_{jj} \end{array}\right] -\left[ \begin{array}{c} \mathbf{\boldsymbol{y}_{-j}^{{\prime}}}\\ \mathbf{V_{j,-j}} \end{array}\right] \mathbf{V}_{-j,-j}^{-1} \left[ \begin{array}{cc} \mathbf{\boldsymbol{y}_{-j}} & \mathbf{V_{-j,j}} \end{array}\right] \\ & =\left[ \begin{array}{cc} \boldsymbol{y}'\boldsymbol{y}-\boldsymbol{y}_{-j}^{\prime}\mathbf{V_{-j,-j}^{-1}}\boldsymbol{y}_{-j} & y_{j}-\boldsymbol{y}_{-j}^{{\prime}}\boldsymbol{V}_{-j,-j}^{-1}\boldsymbol{V}_{-j,j}\\ y_{j}-\mathbf{V_{j,-j}V_{-j,-j}^{-1}\boldsymbol{y}_{-j}} & V_{jj}\mathbf{-V_{j,-j}V_{-j,-j}^{-1}V_{-j,j}} \end{array}\right]. \end{array} $$

Now ***V***_***j,−j***_ in element (2,1) of the above inverse matrix is the vector of covariances between *y*_*j*_ and **y**_−*j*_ and $\mathbf {V_{-j,-j}^{-1}}$ is the inverse of the covariance matrix of **y**_−*j*_. Thus, $\hat {y}_{j}=\boldsymbol {V}_{j,-j}\boldsymbol {V}_{-j,-j}^{-1}\mathbf {y}_{-j}$ is the Best Linear Predictor (BLP) of *y*_*j*_ given **y**_−*j*_, and element (2,1) of (10) is the prediction error of *y*_*j*_. The element (2,2) in (10) is the prediction error variance (PEV) for *y*_*j*_, where $PEV=var(y_{j}-\hat {y}_{j})$. PEV can also be used to calculate theoretical reliability for individual *i* as $1-\frac {PEV_{i}}{V_{jj}}$, and characterizing the distributions of reliability for all the individuals in a dataset has a number of practical applications. Note this allows us to obtain the PEV of every individual and the distribution of these values provide information as to the robustness of genomic predictions across the population of individuals represented in the dataset. This PEV is determined by the genomic variance-covariance matrix and does not depend on **y**. Two different datasets could generate the same PRESS statistic but with different distributions of PEV.

Now, because the permutation matrix **P**_*j*_ is orthogonal, $\mathbf {W}^{-1}=(\mathbf {P}_{j}^{{\prime }}\mathbf {Q}\mathbf {P}_{j})^{-1}=\mathbf {P}_{j}^{{\prime }}\mathbf {Q}^{-1}\mathbf {P}_{j}$, and the elements of **W** ^11^ that are of interest in terms of predicting individual *j* can be obtained directly from **Q** ^−1^ as

11 $$\begin{array}{*{20}l} \mathbf{W}^{11} & =\left[\begin{array}{cc} q^{1,1} & q^{1,(1+j)}\\ q^{(1+j),1} & q^{(1+j),(1+j)} \end{array}\right]. \end{array} $$

It follows that $\hat {e}_{j}$, which is the off-diagonal element of the inverse of the 2×2 matrix **W** ^11^, can be written in terms of **Q** ^−1^ as

12 $$\begin{array}{*{20}l} \hat{e_{j}} & =\frac{-q^{(1+j),1}}{q^{1,1}q^{(1+j),(1+j)}-q^{1,(1+j)}q^{(1+j),1}}, \end{array} $$

where *q* ^*i,j*^ is the element from row *i* and column *j* of **Q** ^−1^. Thus, once **Q** ^−1^ is computed, $\hat {e}_{j}$ for all *j* can be computed using (12), and these values can be used to compute PRESS as $\sum _{j=1}^{n}\hat {e_{j}}^{2}$. To estimate the correlation between the predicted and observed values of *y*_*j*_, the value of $\hat {y}_{j}$ is efficiently obtained as the difference $\hat {y}_{j}=y_{j}-\hat {e}_{j}$.

Now we consider the situation without pre-correcting **y** for *μ*, where E(**y**)=**1** *μ*. Now the mixed model (7) contains both fixed and random effects. Note that the mixed model equations that correspond to this mixed effects model can be derived by treating *μ* as “random” with null mean and large variance. So, let

$$\begin{array}{*{20}l} var\left(\boldsymbol{\beta}^{*}\right) & =\left[\begin{array}{cc} \sigma_{L}^{2} & \mathbf{0^{{\prime}}}\\ \mathbf{0} & I\sigma_{\beta}^{2} \end{array}\right]=\boldsymbol{\Sigma}, \end{array} $$

for sufficiently large value of $\sigma _{L}^{2}$. Then under this assumption, E(**y**)=**0** and $var\left (\boldsymbol {y}\right)=\mathbf {X}^{*}\boldsymbol {\Sigma }\boldsymbol {X}^{*'}+\mathbf {I}\sigma _{e}^{2}=\boldsymbol {V}^{*}$, and thus $\hat {y}_{j}=\boldsymbol {V}_{j,-j}^{*}\boldsymbol {V}_{-j,-j}^{*-1}\mathbf {y}_{-j}$ is the BLP from the random effects rather than mixed effects model of *y*_*j*_ given **y**_−*j*_. This BLP obtained from the model with random *μ* will be numerically very close to the BLUP obtained from the mixed model with fixed *μ*. The **Q** matrix corresponding to the BLP with random *μ* is constructed as $\left [\begin {array}{cc} \mathbf {y'y} & \mathbf {y^{{\prime }}}\\ \mathbf {y} & \mathbf {V}^{*} \end {array}\right ]$ and prediction residuals are obtained as (12).

### Numerical example

Phenotypes ***y*** and genotypes ***X*** at 5 markers for 3 individuals are in Table 1. Assume $\sigma _{\beta }^{2}=\frac {\sigma _{e}^{2}}{10}$ and the overall mean *μ* is the only fixed effect. In LOOCV strategy for MEM and strategy I for BVM, the diagonal elements of ***H*** for MEM and ***C*** for BVM, which are in the denominators of (6) and (9), are in Table 2. The numerators of (6) and (9) are obtained by solving the MME (2) and (8). Then prediction errors are calculated as in (6) and (9) and shown in Table 4. In LOOCV strategy II for BVM, the ***Q*** matrix (Table 3) is constructed using $\sigma _{L}^{2}=1000$, which is sufficiently large relative to $\sigma ^{2}_{e}$ for *μ* to be indistinguishable from a fixed effect with a flat prior. The prediction errors are calculated as (12) and shown in Table 4. The MEM strategy and BVM strategy I gave identical prediction errors and identical PRESS for this numerical example were numerically very close to those from the BVM strategy II.

**Table 1:** Phenotypes and genotypes at 5 markers for 3 individuals used in the numerical example

	M1	M2	M3	M4	M5	Phenotypes
1	1	2	1	2	2	1.97
2	2	1	0	1	1	2.12
3	0	0	2	1	2	–0.62

**Table 2:** Diagonal elements of ***H*** in LOOCV strategy for MEM and ***C*** for BVM

	*j*=1	*j*=2	*j*=3
*H*_*jj*_	0.46	0.51	0.55
*C*_*jj*_	0.46	0.51	0.55

**Table 3:** Q matrix in strategy II for BVM

	1	2	3	4
1	8.75	1.97	2.12	–0.62
2	1.97	1,002.40	1,000.80	1,000.80
3	2.12	1,000.80	1,001.70	1,000.30
4	–0.62	1,000.80	1,000.30	1,001.90

**Table 4:** Prediction errors from different LOOCV strategies (different strategies gave identical prediction errors)

	*j*=1	*j*=2	*j*=3
$\hat {e_{j}}$	1.13	1.21	–2.66

### Simulation to compare efficiency

Two datasets were simulated using XSim [8], where 1,000 offspring were sampled from random mating of 100 parents for 10 non-overlapping generations, to compare the computational efficiencies for naive and efficient strategies using BVM or MEM for LOOCV in GBLUP. Dataset I was simulated with 1,000 observations and 10,000 SNP markers for a *p*≫*n* scenario. Dataset II was simulated with 1,000 observations and 100 markers for a *n*≫*p* scenario. The processor used in the analyses was a 1.4 GHz Intel Core i5 with 4 GB of memory.

For dataset II, efficient MEM is 99 times faster than the naive application (2.979 s versus 0.030 s) (Table 5). All strategies implemented in Julia, a scientific programming language, gave virtually identical prediction accuracies defined as the correlation between **y** and $\hat {\mathbf {y}}$ for each dataset. For dataset I, efficient BVM is 786 times faster than the naive application (3.107 s versus 2,442.59 s) (Table 5).

**Table 5:** Efficiency of alternative LOOCV strategies for GBLUP

	Alternative LOOCV strategies				
	Naive MEM	Naive BVM	Efficient MEM	Efficient BVM I	Efficient BVM II
*n*=1,000; *p*=10,000	9,490.608	2,442.590	105.141	3.107	5.945
*n*=1,000; *p*=100	2.979	169.928	0.030	2.725	0.217

Results are given for the computing time in seconds using naive MEM, naive BVM, efficient MEM, efficient BVM I and efficient BVM II

## Discussion

In genomic prediction, the candidates to be predicted are often offspring that are genotyped but not yet phenotyped. In this situation, LOOCV using all individuals in the training dataset will provide an upper bound for the accuracy of prediction, because ancestors in the training dataset with large numbers of descendants have more accurate predictions than descendants. A better estimate of the accuracy of prediction can be obtained by applying LOOCV to only terminal offspring in the training dataset.

## Conclusions

Efficient strategies for LOOCV in GBLUP are presented in this paper. LOOCV strategy I and II for BVM are more efficient when *p*≫*n*. LOOCV strategy for MEM is more efficient when *n*≫*p*. The accuracy of genomic prediction is often quantified as the correlation between the predicted and observed values of *y*_*j*_, and this correlation can be estimated efficiently using LOOCV strategies. Compared to naive application of LOOCV, which is computationally intensive, LOOCV can be implemented efficiently.
